# FtsH Protease in the Thylakoid Membrane: Physiological Functions and the Regulation of Protease Activity

**DOI:** 10.3389/fpls.2018.00855

**Published:** 2018-06-20

**Authors:** Yusuke Kato, Wataru Sakamoto

**Affiliations:** Institute of Plant Science and Resources (IPSR), Okayama University, Okayama, Japan

**Keywords:** chloroplast development, FtsH protease, photosystem II repair, photosynthesis, post-translational modification (PTM), reactive oxygen species (ROS)

## Abstract

Protein homeostasis in the thylakoid membranes is dependent on protein quality control mechanisms, which are necessary to remove photodamaged and misfolded proteins. An ATP-dependent zinc metalloprotease, FtsH, is the major thylakoid membrane protease. FtsH proteases in the thylakoid membranes of *Arabidopsis thaliana* form a hetero-hexameric complex consisting of four FtsH subunits, which are divided into two types: type A (FtsH1 and FtsH5) and type B (FtsH2 and FtsH8). An increasing number of studies have identified the critical roles of FtsH in the biogenesis of thylakoid membranes and quality control in the photosystem II repair cycle. Furthermore, the involvement of FtsH proteolysis in a singlet oxygen- and EXECUTER1-dependent retrograde signaling mechanism has been suggested recently. FtsH is also involved in the degradation and assembly of several protein complexes in the photosynthetic electron-transport pathways. In this minireview, we provide an update on the functions of FtsH in thylakoid biogenesis and describe our current understanding of the D1 degradation processes in the photosystem II repair cycle. We also discuss the regulation mechanisms of FtsH protease activity, which suggest the flexible oligomerization capability of FtsH in the chloroplasts of seed plants.

## Introduction

**FIGURE 1:**
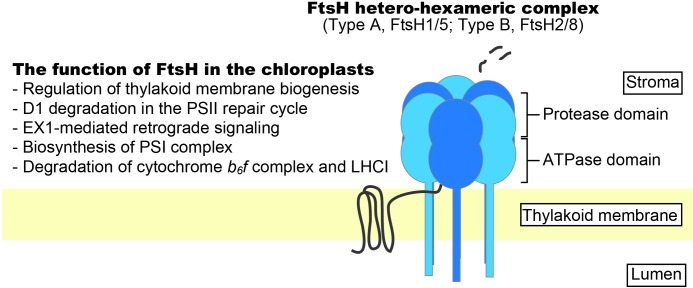
Schematic representation of FtsH protease complex in chloroplasts. Thylakoid FtsH forms a hetero-hexameric structure integrated into the thylakoid membrane. Increasing evidence shows the importance of FtsH in protein quality control in the thylakoid membrane.

Chloroplasts are the essential organelles of seed plants in which photosynthesis takes place. The biogenesis and functions of chloroplasts are dependent on protein homeostasis; therefore, protein quality control, which is orchestrated by the protein synthesis and degradation machinery, is an important process. Chloroplasts originated from a cyanobacterium through endosymbiosis. Thus, the prokaryotic machineries derived from the ancestral cyanobacterium appear to dominate most of the proteolysis mechanisms in chloroplasts. More than 18 kinds of chloroplast proteases have been identified (reviewed by van Wijk, 2015; Nishimura et al., 2016, 2017). Of these, two ATP-dependent proteases, Clp and FtsH, are considered to have major roles in chloroplast protein homeostasis on the basis of their physiological functions. Clp functions in the stroma as housekeeping machinery (reviewed by Clarke, 2012; Nishimura and van Wijk, 2015). In contrast, FtsH protease plays critical roles in the biogenesis of thylakoid membranes and the quality control of thylakoid membrane proteins (**Figure 1**). In this mini review, we describe the functions of FtsH in the quality control of thylakoid membrane proteins and recent findings pertaining to the functions and regulation of FtsH during photooxidative stress.

## Overview of FtsH Proteases in Chloroplasts

FtsH is an ATP-dependent zinc metalloprotease complex that belongs to the AAA (ATPase associated with diverse cellular activities) protease subfamily and is unique because it is membrane anchored (Ito and Akiyama, 2005). The ATPase domain of FtsH functions as an unfoldase and translocates substrates into the degradation chamber through a narrow pore (Krzywda et al., 2002; Niwa et al., 2002). Through this mode of action, FtsH pulls integral membrane proteins out of the membrane and degrades them. According to studies in *Escherichia coli*, more than 20 amino acid residues from either the N- or C-terminal end are required for substrate recognition (Chiba et al., 2000, 2002). *Arabidopsis thaliana* has 12 FtsH-encoding genes. Nine of the corresponding proteins are targeted to chloroplasts and three are localized to mitochondria (Sakamoto et al., 2003). Thylakoid-localized FtsHs possess an N-terminal transmembrane domain and a C-terminal region that extends into the stroma and contains the ATPase and protease domain (Lindahl et al., 1996). FtsHs in the thylakoid membrane form a hetero-hexameric complex consisting of two types of FtsH subunits (type A, FtsH1 and FtsH5; type B, FtsH2 and FtsH8); FtsH2 is the most abundant, followed by FtsH5, FtsH8, and FtsH1 (Sakamoto et al., 2003; Yu et al., 2004, 2005). Mutants lacking the more abundant subunits (i.e., FtsH2 and FtsH5) tend to show stronger phenotypes (e.g., leaf variegation and photosensitivity; see below). Although there is high sequence similarity between the type A and type B subunits, they are integrated into the thylakoid membranes via different pathways; the Sec (secretion) pathway integrates type A subunit FtsH5, whereas the Tat (twin-Arg translocation) pathway integrates type B subunit FtsH2 (Rodrigues et al., 2011). Mutants lacking FtsH2 are known as *yellow variegated2* (*var2*) and show severe leaf variegation (Chen et al., 2000; Takechi et al., 2000). Mutants lacking FtsH5 (*var1*) show weak leaf variegation (Sakamoto et al., 2002), but leaf-variegation phenotypes are not observed in *ftsh8* and *ftsh1* mutants. Meanwhile, the double mutants *ftsh1 ftsh5* and *ftsh2 ftsh8* show an albino-like phenotype, suggesting that both types of subunits are required for active complex formation (Yu et al., 2004, 2005; Zaltsman et al., 2005b). For the Arabidopsis FtsH complex, the stoichiometry of type A:type B subunits was estimated to be 1:2 using a mass spectrometry-based approach (Moldavski et al., 2012). The stoichiometry of subunit types in the FtsH complex in cyanobacteria was shown to be 1:1 (Boehm et al., 2012).

## Functions of FtsH in Chloroplast Biogenesis

Characterization of *var2* mutants has shown that their variegated leaves are composed of green sectors with normal-looking chloroplasts and white sectors with abnormal plastids (Chen et al., 1999; Kato et al., 2007). Accumulation of abnormal plastids in the white sectors is due to arrest in the early stage of plastid differentiation. Notably, normal-looking chloroplasts in the green sectors developed more slowly (Sakamoto et al., 2009). These observations suggest that FtsH is involved in the formation of thylakoid membranes during early chloroplast development. In addition, RNA interference of chloroplast FtsHs in *Nicotiana tabacum* showed a collapse of thylakoid membranes during the late stages of leaf development, suggesting that FtsH also plays a crucial role in the maintenance of thylakoid membranes (Kato et al., 2012a). Based on the correlation between the level of the FtsH complex and the degree of leaf variegation, a threshold model in which total FtsH levels define the fate of plastids during leaf development has been proposed (Chen et al., 2000; Yu et al., 2004; Zaltsman et al., 2005a; Miura et al., 2007). Furthermore, complementation of *var2* by site-directed mutagenesis of FtsH2 demonstrated that not all catalytic sites are required for the adequate development of thylakoid membranes (Zhang et al., 2010). Although the exact role of FtsH in thylakoid development remains to be elucidated, it has also been suggested that FtsH acts as a scaffold protein during thylakoid development. On the other hand, multiple lines of evidence demonstrate that reduction in protein biosynthesis in plastids suppresses leaf variegation (Miura et al., 2007; Yu et al., 2008; see details. Putarjunan et al., 2013 for a review). The balance between protein biosynthesis and FtsH function during leaf development seems to be crucial for chloroplast differentiation.

## Protein Quality Control in the Photosystem II Repair Cycle

**FIGURE 2:**
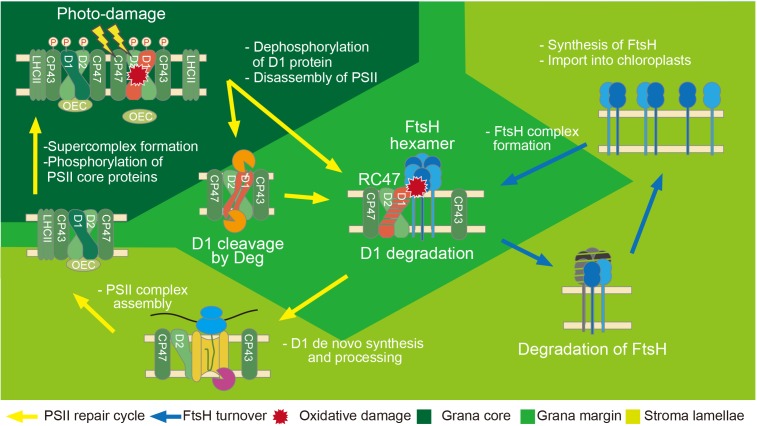
Model of PSII repair and regulation of FtsH activity in chloroplasts. In chloroplasts, photodamaged D1 migrates from the grana stacks to the grana margins, which is the location of D1 degradation in the PSII repair cycle. Dephosphorylation of PSII core proteins by chloroplast PP2C phosphatase and partial disassembly of the PSII complex occur prior to the degradation process. D1 degradation is mainly conducted by FtsH (fundamental degradation), and endopeptidic cleavages by Deg proteases facilitate the effective degradation by FtsH in photoinhibitory conditions. Newly synthesized D1 is processed at its C-terminus by CtpA peptidase. Repaired PSII migrates to the grana core to form functional PSII. Phosphorylation of PSII core proteins is carried out by STN8 kinase. On the other hand, the PSII repair cycle might require proper FtsH turnover in chloroplasts. When FtsH has access to damaged PSII, FtsH concomitantly suffers from oxidative damage induced by ROS. The damaged FtsH should be repaired by proper turnover. Newly synthesized FtsH proteins are imported into chloroplasts and subsequently integrated into the thylakoid membrane. FtsH forms functional complexes in the grana margin. The post-translational modification in FtsH might regulate protease activity and/or complex formation.

The main function of FtsH in chloroplasts is protein quality control during photosynthesis. Light energy is required for the initial step of photosynthesis but it simultaneously causes unavoidable damage to the photosystem II (PSII) protein complex, in particular to the reaction center protein D1 (Murata et al., 2007). Photodamaged D1 needs to be removed specifically by proteolysis and replaced by newly synthesized D1 to maintain photosynthetic activity. This sophisticated repair system that photosynthetic organisms have developed is called the “PSII repair cycle,” and it consists of a sequential process of (i) light-induced damage to the reaction center protein D1, (ii) partial disassembly of the PSII complex to expose damaged D1, (iii) proteolysis of damaged D1, and (iv) de novo synthesis of D1 and reassembly of functional PSII (**Figure 2**). FtsH is involved in the proteolysis of damaged D1 in the PSII repair cycle (Nixon et al., 2010). This fundamental process is conserved in various photosynthetic organisms and is crucial for avoiding photoinhibition caused by the accumulation of photodamaged PSII. Indeed, Arabidopsis mutants lacking FtsH show increased sensitivity to high-light stress, which is indicative of accelerated photoinhibition (Bailey et al., 2002; Sakamoto et al., 2002, 2004).

Thus far, two proteolytic pathways involving FtsH for removing damaged D1 have been described. The first pathway is processive D1 degradation, which is carried out predominantly by FtsH (Lindahl et al., 2000; Bailey et al., 2002; Kato et al., 2009). D1 has five transmembrane domains and a stroma-exposed N terminus, at which FtsH might initiate processive proteolysis. Studies using cyanobacteria mutants and transgenic tobacco plants with truncations at the N-terminus of D1 showed that the N-terminus is required for D1 degradation (Komenda et al., 2007; Michoux et al., 2016). In addition, post-translational modifications (PTMs) at the N-terminus seem to regulate D1 degradation by FtsH. Earlier studies reported that an N-terminal threonine residue of D1 is reversibly phosphorylated in a light-dependent manner (Aro et al., 1992), and its dephosphorylation is a prerequisite for its degradation (Koivuniemi et al., 1995; Rintamaki et al., 1996). The fact that phosphorylation of D1 increased more in mutants lacking FtsH in a light-dependent manner suggests an interaction between D1 phosphorylation and processive degradation by FtsH (Kato and Sakamoto, 2014). While more phosphorylation prevents FtsH from degrading D1 efficiently, it appears to accelerate the alternative degradation pathway mediated by Deg proteases concomitantly (see below). This coordinated D1 degradation was shown to be compromised in the mutant lacking STN8 kinase that phosphorylates D1, when combined with *var2*. The N-terminus of D1 is also processed by methionine aminopeptidases, and enzymatic removal of the N-terminal-initiating methionine seems to be required for recognition of the N-terminus by FtsH (Adam et al., 2011).

The second D1 degradation pathway consists of the cooperative action of FtsH and Deg proteases in photoinhibitory conditions. Deg family members are ATP-independent serine proteases and are localized to both the stromal and luminal sides of thylakoid membranes (Schuhmann and Adamska, 2012). Endopeptidic cleavage of D1 by Deg proteases seems to facilitate D1 degradation by creating additional recognition sites for FtsH (Haußühl et al., 2001; Kapri-Pardes et al., 2007; Sun et al., 2007, 2010; Knopf and Adam, 2018). Evidence for this cooperative D1 degradation pathway comes from the increased accumulation of D1 cleavage fragments in Arabidopsis mutants lacking FtsH (Kato et al., 2012b). Accumulation of D1 cleavage fragments has also been observed in *Chlamydomonas reinhardtii ftsh* mutants (Malnoë et al., 2014). However, the degradation rate of D1 protein in a *deg* triple mutant in the cyanobacteria *Synechocystis* sp. PCC 6803 is similar to that in wild-type controls, suggesting that the contribution of Deg proteases is less important in cyanobacteria (Barker et al., 2006). A detailed analysis of D1 cleavage fragments in Arabidopsis suggests that fragmentation is initiated by Deg proteases that cleave the luminal loop connecting the C and D transmembrane helices of D1. This hypothesis might be reinforced by the fact that D1 fragmentation is enhanced by primary damage to the Mn-cluster, which might induce dissociation of the PSII oxygen-evolving complex and accelerate the access of Deg proteases to damaged D1 (Kato et al., 2015). Accelerated D1 degradation through D1 fragmentation appears to cause a simultaneous increase in reactive oxygen species (ROS) levels. This relationship could be explained by cytotoxicity of D1 cleavage fragments, which may retain chlorophyll that can still absorb light energy. Although this energy could not be used for photosynthesis, it could be transferred to oxygen, resulting in the generation of ROS. Cooperative D1 degradation is considered to be an escape pathway that occurs in photoinhibitory conditions (Kato and Sakamoto, 2014).

## Oxidative Stress and Retrograde Signaling

Mutants lacking FtsH generate high levels of ROS as a result of the accumulation of damaged PSII in chloroplasts (Kato et al., 2009). Because ROS have been implicated as second messengers that transduce retrograde signals from chloroplasts to the nucleus (Laloi et al., 2006), we were interested in examining gene expression in mutants lacking FtsH. However, our attempt to do this via microarray analysis showed that there was no obvious up-regulation of ROS-related genes in cells suffering from photooxidative stress (Miura et al., 2010). This unexpected result suggests that ROS from photodamaged PSII do not act as second messengers or that the signaling might be inhibited by the loss of FtsH. Notably, a recent study by Wang et al. (2016) reported the involvement of FtsH in an EXECUTER1 (EX1)-mediated retrograde signaling pathway activated by singlet oxygen (^1^ O_2_). EX1 was originally identified through a suppressor screen of *fluorescent* (*flu*) mutants, which generate ^1^ O_2_ in chloroplasts upon a dark-to-light shift (Lee et al., 2007; Przybyla et al., 2008). In the *flu* mutant, an ^1^ O_2_ burst resulting from the abnormal accumulation of a chlorophyll precursor causes the degradation of EX1 and eventually leads to programmed cell death. In this process, FtsH degrades EX1, which might be oxidized by ^1^ O_2_ (Wang et al., 2016). Because the inactivation of FtsH in *flu* mutants prevents ^1^ O_2_ signaling and related gene expression (Wang et al., 2016; Dogra et al., 2017), the degradation of EX1 by FtsH is necessary for the retrograde signaling pathway. The mechanisms by which EX1 degradation products act as retrograde signaling molecules remains to be investigated.

## Other Biological Functions of FtsH

FtsH is involved in the degradation and assembly of several thylakoid proteins other than D1. An early *in vitro* study suggested that FtsH participates in the degradation of unassembled Rieske Fe-S protein, a component of the thylakoid-bound cytochrome *b_6_f* complex that may undergo a conformational change upon absorption of light energy (Ostersetzer and Adam, 1997). A later study in *Chlamydomonas* showed that the cytochrome *b_6_f* complex is degraded by FtsH in conditions of sulfur or nitrogen starvation, indicating that FtsH plays a role in protein quality control during nutrient deficiency (Malnoë et al., 2014; Wei et al., 2014). On the other hand, Järvi and coworkers showed that FtsH functions in the biosynthesis of PSI (Järvi et al., 2016). Furthermore, the low levels of FtsH in Arabidopsis *ftsh* mutants seem to affect the entire photosynthetic electron transfer chain. Analysis of chlorophyll fluorescence implies that mutants lacking FtsH exhibit high rates of cyclic electron transfer around photosystem I (PSI), which results in a higher non-photochemical quenching value (Järvi et al., 2016). Additionally, *Chlamydomonas* FtsH plays a role in degrading the light-harvesting chlorophyll a/b-binding proteins associated with PSI (Bujaldon et al., 2017). How FtsH is involved in the early assembly of PSI complexes remains unknown; however, these results expand our understanding of the physiological function of FtsH in thylakoid membranes.

## Regulation of FtsH Protease Activity

Several important questions remain to be answered, as to how the protease activity of FtsH in chloroplasts is regulated. *E. coli* FtsH hexamers interact with other membrane protein complexes composed of the prohibitin-like proteins HflK and HflC (Kihara et al., 1996; Saikawa et al., 2004). Increasing evidence suggests that prohibitin-like proteins act as a negative regulator in the selection of FtsH substrates (Kihara et al., 1996, 1998). Prohibitin homologs (PHBs) have also been implicated in regulating FtsH activity in other bacteria, yeast, and plant mitochondria (Steglich et al., 1999; Piechota et al., 2010). In photosynthetic organisms, however, supercomplexes consisting of thylakoid FtsH and PHBs have been reported only in cyanobacteria (Boehm et al., 2012). Although FtsH-containing supercomplexes have been observed in *Chlamydomonas* chloroplasts (Wang et al., 2017), PHBs have not been found in the chloroplasts; therefore, the composition of these supercomplexes remains unknown. In Arabidopsis, blue native polyacrylamide gel electrophoresis analysis of thylakoid membranes showed that most FtsH proteins migrated in small complexes, likely in the form of dimers, whereas a small amount was present as a larger complex, at the position where a functional complex would be expected (Takabayashi et al., 2017). Yoshioka et al. (2010) suggested that smaller FtsH complexes are mainly present in the stroma-exposed thylakoid, whereas the large functional complex is detected in PSII-enriched thylakoid membranes, including grana margins where D1 degradation occurs during the PSII repair cycle. These results suggested the flexible oligomerization capability of FtsH proteins and the existence of different mechanisms for the regulation of FtsH activity in the chloroplasts of seed plants. Instead of forming supercomplexes with PHBs, an interesting hypothesis is that FtsHs in seed plants might transiently form a functional hexameric complex to fulfill its degradation function. A recent proteomics analysis demonstrated that thylakoid FtsH proteins themselves show a higher turnover rate than other chloroplast proteases (Li et al., 2017). Given that damaged PSII complexes are a potential site of ROS generation, FtsH might suffer from oxidative damage as well and require its own quality control by means of its fast turnover (**Figure 2**). Supporting this possibility, degradation of FtsH was suggested to be accelerated during high-light stress (Zaltsman et al., 2005a). Sinvany-Villalobo et al. (2004) reported that upon high-light exposure, some *FtsH* genes are dramatically upregulated at the mRNA level (e.g., *FtsH8*), whereas FtsH protein levels showed only modest increase. Overall, these data implicate a fast turnover rate of FtsH, which should be further investigated in the future works.

In addition to the possible turnover of FtsH during light stress, PTMs seem to contribute to its regulation. Thus far, two PTMs are thought to regulate FtsH function. In Arabidopsis, phosphorylation of FtsH in the chloroplasts was suggested by a proteome study on calcium-dependent protein phosphorylation (Reiland et al., 2009; Stael et al., 2012; Roitinger et al., 2015). Whether phosphorylation controls FtsH activity remains unclear, and we are currently testing this possibility by means of site-directed mutagenesis of predicted phosphorylation sites. On the other hand, a recent study in *Chlamydomonas* showed that the formation of intermolecular disulfide bridges promoted the oligomerization of FtsH (Wang et al., 2017). Of note, these results suggested that the proteolytic activity of FtsH could be regulated by the thylakoid redox state, because the formation of disulfide bridges in chloroplasts is controlled mainly by the thioredoxin system in the stroma and thylakoid membrane.

## Perspectives and Remaining Questions

A recent study in cyanobacteria suggests that Thylakoid formation 1 (THF1) protein, which seems to positively affect the accumulation of FtsH, physically interacts with FtsH (Beèková et al., 2017). Together, increasing evidence indicates that the stability and complex formation of FtsH are key to understanding the details of FtsH function. Another remaining question is the issue of substrate recognition. Focusing on the PSII repair cycle, selective D1 recognition by FtsH after disassembly of CP43, the core antenna protein of PSII, from the damaged PSII complex might be important for enhancing photosynthesis in plants by improving the PSII repair. Krynická et al. (2015) showed that the accessibility of FtsH to the D1 protein is crucial for its selection of substrates in the PSII complex. For example, oxidative modification of the PSII reaction center may be associated with D1 degradation. Recent progress in mass spectrometry has enabled the identification of specific photooxidative protein modifications in PSII core proteins (Dreaden et al., 2011). Future research in this area will uncover the relationship between photooxidative protein modifications and proteolysis by FtsH.

## Author Contributions

All authors listed have made a substantial, direct and intellectual contribution to the work, and approved it for publication.

## Conflict of Interest Statement

The authors declare that the research was conducted in the absence of any commercial or financial relationships that could be construed as a potential conflict of interest.
